# Relationship of Absolute and Relative Lower-Body Strength to Predictors of Athletic Performance in Collegiate Women Soccer Players

**DOI:** 10.3390/sports6040106

**Published:** 2018-09-29

**Authors:** Emily Andersen, Robert G. Lockie, J. Jay Dawes

**Affiliations:** 1 Department of Health Sciences, University of Colorado Colorado Springs, Colorado Springs, CO 80918, USA; ekolsevi@uccs.edu; 2 Department of Kinesiology, Cal State University-Fullerton, Fullerton, CA 92831, USA; rlockie@fullerton.edu

**Keywords:** maximum strength, speed, agility, change of direction speed, power, testing

## Abstract

The purpose of this study was to investigate the relationships between absolute and relative lower-body strength on predictors of athletic performance among Division II collegiate women’s soccer players. Archived pre-season testing data for seventeen (*n* = 17) female National Collegiate Athletics Association (NCAA) Division II soccer players were analyzed, including: vertical jump, 3RM back squat, 505-agility, modified T-test, 10 m and 30 m sprint, and 20 m multistage fitness test (20 m MSFT). Relative strength was calculated based on the estimated 1RM back squat divided by the athlete’s body mass. Significant correlations were discovered between absolute lower-body strength and 505-agility (Right: *r* = −0.51, *p* < 0.05; Left: *r* = −0.59, *p* < 0.05), modified T-test (*r* = −0.55, *p* < 0.05), 10 m and 30 m (*r* = −0.59, *p* < 0.05; *r* = −0.54, *p* < 0.05), and sprint performance. Relative lower-body strength showed significant correlations with vertical jump (*r* = 0.54, *p* < 0.05), 505-agility (Right: *r* = −0.58, *p* < 0.05; Left: *r* = −0.67, *p* < 0.01), modified T-test (*r* = −0.75, *p* < 0.01), 10 m and 30 m (*r* = −0.59 *p* < 0.05; *r* = −0.67, *p* < 0.01), and the 20 m MSFT (*r* = 0.58, *p* < 0.05). These results indicate that strength and conditioning coaches should emphasize the development of absolute and relative lower-body strength with their players to improve power, agility, and speed performance.

## 1. Introduction

Soccer is one of the most widely played sports in the world and continues to grow in popularity with female athletes of all ages [1,2,3]. This sport requires repetitive, high-intensity bouts of maximal exercise, tactical, technical, and physical skills to be successful [2,3,4]. In a regular match, athletes frequently perform quick sprints lasting less than 6 s for every 90 s of play, between 1200–1400 high-intensity changes of direction, all at heart rates up to 80–90% of their age-predicted estimated maximum heart rate [2,3,4]. Based on these demands achieving balance between strength, power, and endurance capabilities is critical for success over the course of a 90+ minute game [2]. However, at this time there is a paucity of research related to how these factors relate to one another among female collegiate athletes, especially at the NCAA Division II level. This information may be useful to strength and conditioning professionals when attempting to enhance athletic performance within this population. 

Lower-body strength may play a significant role in a soccer player’s on-field performance [5]. Previous investigations have explored the relationships between absolute strength and several predictors of athletic performance in male soccer players [6,7]. For example, De Hoyo et al. [6] and Hammami et al. [7] discovered that maximal strength, as measured by the one-repetition maximum (1RM) back squat, was highly correlated with improved sprint performance, change of direction speed, and vertical jump height in adolescent male soccer players. Similarly, Stølen et al. [2], Styles et al. [8], Turner et al. [3], and Wisløff et al. [9,10] reported significant correlations between 1RM back squat strength and sprint performance, vertical jump height, and aerobic endurance in elite male soccer players. Additionally, relative strength has been shown to strongly correlate with both sprint and change of direction speed among male athletes in a variety of sports. In a study by Keiner et al. [11], the researchers discovered a high correlation between relative strength and change of direction ability among adolescent male soccer players. Styles et al. [8] also reported a relationship between relative strength and sprint performance for professional male soccer players. Research by McBride et al. [12] found that relative strength highly correlated with improved sprint time for Division I football players. However, at this time, the specific relationships between strength and predictors of on-field performance have not been thoroughly explored among female soccer players [13]. 

Currently, there is limited research on the relationships between selected predictors of soccer performance and strength among female soccer athletes. Brooks et al. [13] found that isokinetic lower-body strength was significantly related (r = 0.93, *p* < 0.05) to ball velocity among 22 Division I female soccer players. Furthermore, low to moderate relationships were seen between 1RM back squat performance and the 40 yard (r = 0.032, *p* < 0.05) and 100 m (r = 0.46, *p* < 0.05) sprints. A moderate relationship was also discovered between estimated VO_2_ maximum and 1RM back squat strength (r = 0.48, *p* < 0.05). However, the relationship between relative strength and these variables was not addressed in this study. Nimphius et al. [14] did find that relative strength correlated with speed and change of direction capabilities for female softball players. While relative strength has been shown to be important in soccer for controlling body mass during tactical movements on the field and during acceleration and deceleration [2,10], the relationships between relative strength and other predictors of on-field performance have not been fully explored in female athletes at different levels of play.

Several studies have found that the lower-body power is correlated with sprint performance and change of direction speed [14,15]. As such, improving power is a primary focus of many strength and conditioning programs [16]. According to Dawes and Lentz [16], there are several ways to improve power to improve speed. One recommendation by these authors is to improve both absolute and relative strength through a combination of lower-body resistance training, weightlifting and plyometric training. The authors posit that by improving force production capabilities via increases in lower-body strength, and the combination of explosive training, that sprint performance may be improved. Based on the repeated bursts of high intensity sprinting observed in soccer it makes sense that increased strength may yield greater power capabilities and improve speed performance. However, relationship between lower-body strength and aerobic performance among female soccer players is unclear. Greater strength may help reduce the negative impact of fatigue over the course of a 90 min match, since an athlete would potentially be required to use less energy to perform the same task [2,3,5,7]. This may be especially relevant based on the eccentric demands associated with the need to frequently change directions and decelerate during completion [2,5].

Currently, the majority of research related to strength and soccer performance has been conducted on male soccer athletes [17]. Thus, there is a significant gap in the literature as it relates to the impact of strength on measures of performance in female soccer athletes. This information may be useful to strength and conditioning professionals when developing a comprehensive training program to meet the needs of this specific population. Therefore, the purpose of this study was to assess if absolute and relative lower-body strength correlated with pre-season performance variables in women’s NCAA Division II collegiate soccer players. The researchers hypothesized that both absolute and relative strength would relate to all the performance tests used in this study.

## 2. Materials and Methods

### 2.1. Participants

Seventeen (*n* = 17) Division II collegiate women’s soccer players (age = 19.7 + 1.2 years; height = 166.4 + 7.8 cm; body mass = 64.3 + 7.9 kg) participated in normal pre-season testing. Written consent to use testing data was obtained from the athletes at the beginning of the academic year via the athletic department at the university in which this research occurred. Due to the archival nature of this data, the study qualified for exempt review via the Institutional Review Board (IRB) for human subjects at the University of Colorado, Colorado Springs.

### 2.2. Protocol

The pre-season fitness testing took place over two days, with 48 h rest between testing sessions. Each session included a ten-minute standardized dynamic warm-up consisting of callisthenic type exercise, and light running activities. Participants were then provided full instruction on how to perform each test. Instructions for each test ranged between 1–2 min. Athletes were allowed up to two trials practice allowed if necessary. During the first testing session the following tests were performed in the order listed in the university weight room: vertical jump (VJ) and the three-repetition maximum back squat (3RM). For the second testing session, the following drills were performed in the order listed: 505-agility (505); modified T-test (Mod T); 10 m and 30 m sprints; and the 20 m Multistage Fitness Test (20 m MSFT). All speed and agility testing was performed on a turf soccer field.

### 2.3. Body Mass

Height (cm) and body mass (kg) were measured as anthropometric data, on a doctor’s beam scale (Cardinal; Detecto Scale Co, Webb City, MO, USA) [15,18]. Body mass was used in this study to determine the relative strength of each athlete.

### 2.4. Vertical Jump

Lower-body power was measured on a Just Jump Mat (Just Jump, Pro Biotics Inc., Hunstville, AL, USA) with a countermovement vertical jump [18]. This test, described by Dawes et al. [18], allowed three separate trials and retained the best one for analysis. Each jump occurred with approximately 10 s between trials, which provided players enough time to recover. The best score was recorded to the nearest tenth of an inch, then converted to centimeters for analysis (i.e., inches × 2.54).

### 2.5. 3RM Back Squat

Maximal lower-body strength was assessed via the 3RM back squat test. Initial training loads for this assessment were prescribed using self-reported data for the 3RM that was performed and recorded by the athletes during their summer training programs three weeks prior to this testing session. This information was used to estimate their 1RM and estimate the loads that would be used during the warm-up protocol for this test. As described by Dawes et al. [18], each athlete was to warm up with the following protocol: 5 repetitions at 30% their one-repetition maximum (1RM), 8 repetitions at 50% 1RM, 6 repetitions at 60% 1RM, 5 repetitions at 70% 1RM, and 3 repetitions at 80% 1RM, resting 3 min between each trial. Once the final 3RM was performed, it was converted via previously established equations to a predicted 1RM for the individual’s absolute strength measure [18]. Relative strength was individually calculated for each athlete with the following equation: (kilograms lifted)/(body mass).

### 2.6. 505-Agility

The 505-agility test was a pre-determined change of direction test measuring the athlete’s ability to make a 180-degree turn. The procedure, explained by Shepard et al. [19], describes the athletes building up speed for 10 m and upon passing the electronic timing system (TC-System, Brower Timing Systems, Draper, UT, USA) [15], sprinting 5 m, making a 180-degree turn, and sprinting 5 m back through the timing gate to complete the test. A total of two trials for each leg were recorded and the best score from each side was retained for analysis.

### 2.7. Mod T

This assessment was used to determine agility and change of direction with multiple locomotion actions (i.e., sprinting, backpedaling and side shuffling). From the protocol described by Sassi et al. [20], athletes were instructed to begin at the starting line then sprint forward 5 yards to the center cone and touch this cone with the right hand. Upon touching the center cone the athlete immediately shuffled 2.5 yards to their left and touched the corresponding cone with the left hand before shuffling 5 yards to the right and touching a cone with the right hand. The athlete then shuffled 2.5 yards back to the center cone, touched this cone with the left hand, then backpedaled to the starting line to complete the test. The difference in protocols for the normal T-test and the modified T-test was also described by Sassi et al. [20] stating the modified test uses half the distances from the original test, requiring athletes to only cover a total distance of 20 m, rather than 40 m.

### 2.8. 10 m and 30 m Sprint

Linear sprint velocity was measured using an electronic timing system (TC-System, Brower Timing Systems, Draper, UT, USA) [18]. The protocol, described by McFarland et al. [15], had timing gates set up in a straight line on the turf to capture both acceleration (10 m) and maximal velocity (30 m) for analysis. The best of three trials was recorded and rounded to the nearest tenth of a second.

### 2.9. 20 m MSFT

This multistage fitness test was performed to determine aerobic fitness. A line of cones was placed 20 m away and parallel from a solid white line on the turf field. The athletes were instructed to run back and forth between the cones, touching the line with their foot on each side, in pace with an audible “beep” sound from the pre-recorded testing application [18]. The test initially started at a speed of 8.5 km/h and increased 0.5 km/h with each stage [21]. Athletes were warned if they did not touch the line on or before the subsequent “beep” the test would be terminated. The test was terminated when athletes missed the line on two consecutive beeps. The total number of shuttles completed prior to the final missed shuttle level was recorded for analysis.

### 2.10. Statistical Analysis

Data was analyzed using IBM SPSS statistics (Version 24.0; IBM Corporation, New York, NY, USA). Descriptive statistics (mean ± SD) were calculated for each variable. Pearson’s correlation coefficient was used to relate the absolute and relative lower-body strength measures to scores on the performance tests. Statistical analysis was set at the a priori *p* ≤ 0.05 level. The strength of each correlation value were as follows: 0 to 0.30, or 0 to −0.30 was considered low; 0.31 to 0.49, or −0.31 to −0.49 moderate; 0.50 to 0.69, or −0.50 to −0.69 large; 0.70 to 0.89, or −0.70 to −0.89 very large; and 0.90 to 1.0, or −0.90 to 1.0 a near perfect correlation [22]. 

## 3. Results

Descriptive data for the subjects is shown in Table 1. Significant large correlations were found between absolute strength and 505, Mod T, and 10 m and 30 m sprint intervals. Significant large to very large correlations were found between relative strength and VJ, 505, Mod T, 10 m and 30 m sprint intervals, and 20 m MSFT. There were no statistically significant correlations found between absolute strength and VJ or the 20 m MSFT. These results are presented in Table 2.

## 4. Discussion

The main purpose of this retrospective study was to determine if there was a relationship between absolute and relative lower-body strength and performance tests used to predict soccer performance among Division II female soccer players. The main finding of this study were that significant correlations did exist between lower-body strength and performance, especially relative strength. Relative strength had large to very large correlations with each of the performance variables tested. These results suggest that improving the absolute and relative lower-body strength of collegiate female soccer players can be beneficial to their performance.

In the current study, significant correlations between absolute strength and change of direction ability and linear speed were found in this study. These data support previous research conducted with male soccer players. Absolute strength has been found to be highly correlated with increased sprint performance [2,3,6,7,8,9], as well as change of direction speed [3,7] among male soccer players. The results of this study suggest developing absolute lower-body strength may improve both sprint and change of direction speed among collegiate female soccer players.

The current study also identified significant correlations between relative strength and each of the performance variables tested. This supports the findings of previous research that have reported significant correlations between relative strength, change of direction ability, and sprint speed among young male soccer athletes, football players, and female softball players [8,11,12,14]. Interestingly, the current study found strong correlations with relative strength, vertical jump, and aerobic endurance. This further supports the notion that improving relative strength among female soccer players may help improve on-field performance to a larger extent than focusing on the development of absolute strength alone.

With that said, significant correlations between absolute strength, vertical jump height and aerobic endurance were not discovered. This contrasts with the findings of De Hoyo et al. [6] that reported significant improvements vertical jump performance after performing a full-squat training program. Stølen et al. [2], Turner et al. [3], and Wisløff et al. [9,10] all reported significant correlations between maximal strength and an increase in vertical jump and aerobic endurance performance in male soccer athletes. These findings are also in contrast to Brooks et al. [13] that found a moderate relationship between absolute strength and vertical jump height and a low relationship between absolute strength and aerobic capacity. Based on the results these investigations it would appear that improving absolute strength might be of most benefit if it also enhances relative strength in this population. For this reason, strength and conditioning professionals should emphasize improving an athlete’s strength to body mass ratio [16].

The current investigation adds to the previous research, showing positive correlations between absolute and relative lower-body strength and performance variables among Division II women’s soccer players. However, this study is not without limitations. This study used archived data for one season and one team. Data related to the individual athletes playing experience was unavailable. This may be a useful variable to analyze in future research, as athlete’s years of experience may influence the outcomes of interest in the present study. Additionally, the focus of this study was to analyze the relationships between strength, power, and speed among a Division II women’s soccer team. Another limitation is the small sample size, only recognizing testing for 17 individuals. Further research should investigate each year to determine strengths and weaknesses of training. Over time, data could also be investigated correlating performance to on-field performance/success.

## 5. Conclusions

Based on the results of this study it appears that when developing strength and conditioning programs for collegiate women’s soccer players, coaches should focus on developing training programs that specifically target improving both absolute and relative strength. While relative strength appears to have the greatest impact on predictors of athletic performance in collegiate women’s soccer, the development of absolute strength should enhance relative strength as long as significant increases in body mass do not occur. Furthermore, the development of strength may have somewhat of a ceiling effect within the sport of soccer, in which an emphasis on developing more strength may actually detract from other areas of training (i.e., speed, agility, power training) [1,3,5,15,20]. Therefore, soccer athletes should seek to develop the optimal level of strength to be successful in their sport, rather than simply trying to become perpetually stronger. 

## Figures and Tables

**Table 1:** Descriptive statistics of participants and overall team averages for each performance variable.

Variable	Number of Participants	Team Average ± SD *
Age (years)	17	19.7 ± 1.2
Height (cent)	17	166.4 ± 7.9
Body mass (BM) (kgs)	17	64.3 ± 7.9
Vertical Jump (cent)	17	45.72 ± 2.95
505 Right (s)	17	2.64 ± 0.12
505 Left (s)	17	2.68 ± 0.12
Mod T (s)	17	6.30 ± 0.40
10 m (s)	17	2.10 ± 0.12
30 m (s)	17	5.16 ± 0.25
20 m MSFT (shuttle #)	16	68.81 ± 12.15
1RM Back Squat (kgs)	17	73.80 ± 9.98
Relative Squat (squat kg/BM kg)	17	1.16 ± 0.18

* Team averages and standard deviations are rounded to the nearest hundredth.

**Table 2:** Absolute and relative lower-body strength in correlation to pre-season performance testing.

Performance Tests	VJ	505 R	505 L	Mod T	10 m	30 m	20 m MSFT
Absolute Strength							
Pearson Correlation	0.331	−0.512 *	−0.594 *	−0.550 *	−0.589 *	−0.539 *	0.468
Significance	0.194	0.036	0.012	0.022	0.013	0.026	0.068
Relative Strength							
Pearson Correlation	0.546 *	−0.584 *	−0.675 **	−0.752 **	−0.593 *	−0.669 **	0.576 *
Significance	0.023	0.014	0.003	0.000	0.012	0.003	0.019

* = Significance at ≤0.05 level; ** = Significance at 0.01 level.
